# Characterization of the complete mitogenome of the Hongyuan Yak *Bos grunniens* (Artiodactyla: Bovidae) and its phylogenetic analysis

**DOI:** 10.1080/23802359.2020.1787255

**Published:** 2020-07-06

**Authors:** Lichun Jiang, Xia Zhu, Jing He, Ximin Fu, Xiwen Chen, Bingxiu Wu, Tian Sun, Jing Luo

**Affiliations:** a Key Laboratory for Molecular Biology and Biopharmaceutics, School of Life Science and Technology, Mianyang Normal University, Mianyang, P.R. China; b Ecological Security and Protection Key Laboratory of Sichuan Province, Mianyang Normal University, Mianyang, P.R. China

**Keywords:** *Bos grunniens*, Bovidae, mitochondrial genome, genome organization, phylogenetic relationships

## Abstract

The Hongyuan breed Yak (*Bos grunniens*) belongs to a member of t the subfamily Bovinae. We provide a complete mitogenome of *B. grunniens* and analyze its phylogenetic relationship with other related species. Its mitogenome is a circular molecule with 16,322 bp in size, including 13 protein coding genes, 22 tRNA genes, 2 rRNA genes, and a non-coding control region (D-loop, CR) that are conserved in most Bovidae mitogenomes. The total base composition of the *B. grunniens* mitogenome is 33.67% A, 27.29% T, 25.84% C, and 13.20% G. The gene composition, structure and the arrangement for *B. grunniens* are similar to those of most other Bovidae species. Phylogenetic analysis of mitochondrial genomes of 30 close species with Bayesian inference and maximum likelihood based on 13 protein-coding genes indicated that *B. grunniens* breed Hongyuan is more closely related to *B. grunniens* breed Qinghai Plateau than to *B. grunniens* breed Xuedong and *B. grunniens* breed Maiwa. The complete mitogenome of *B. grunniens* breed Hongyuan provides a potentially useful resource for further exploration of the taxonomic status and phylogenetic relationships of Bovinae and related species.

Yak is unique and important biological species breeds in the Qinghai-Tibet plateau, the pamir and Himalayan plateau, which can provide most important production and living materials for local aborigines in these areas (Chu et al. 2016; Fu et al. 2019). The development of yak industry related to regional social and economic development and people’s life. The Yak breed (*Bos grunniens*) from Hongyuan County belongs to a member of the subfamily Bovinae. It lives at the boundary of the Qinghai-Tibet Plateau, China, which has good adaptability to the plateau extreme environment (high altitude, hypoxia, high ultraviolet, etc.) and diseases resistance (Hu et al. 2012; Ma et al. 2012). However, fewer mitochondrial information about the Hongyuan Yak breed could be used to compare its genetic evolution relationship with other yak breeds. To better understand the mitogenomic characteristics, phylogeny and evolution of the Bovidae, the whole mitogenome sequence of the yak breed was characterized and annotated to provide useful information to feature analysis and system evolution research.

The muscle samples were collected from Hongyuan County, Aba Prefecture, Sichuan province, China in November 2019 (102°19′44.55″E, 32°38′59.84″N) and immediately preserved in 95% ethanol and stored at −80 °C until use. The voucher specimens were stored in the Key Laboratory for Molecular Biology and Biopharmaceutics from Mianyang Normal University (LC2019112008). The sample was extracted by phenol-chloride method (Sambrook and David 2001). We employed Long-and-Accurate PCR methods to amplify the whole mitogenomic region of *B. grunniens* with the self-designed and reported primers (Bao et al. 2016; Gu et al. 2007; Wang et al. 2010). The whole sequence of the *B. grunniens* mtDNA was determined and deposited to the GenBank DNA databases under accession number MT162465.

The complete mtDNA of *B. grunniens* is a circular molecule with 16,322 bp in size and include 37 genes, 13 protein-coding genes (PCGs), 2 ribosomal RNA genes (12S rRNA and 16S rRNA), 22 tRNA genes and a control region (D-loop). The base composition of the whole mitogenome is 33.67% A, 27.29% T, 25.84% C, and 13.20% G. The gene composition and the arrangement for *B. grunniens* are similar to those of most other Bovidae species (Cheng et al. 2011; Douglas et al. 2011; Hassanin et al. 2012; Tu et al. 2014; Guo et al. 2016). Most genes are encoded on the heavy strand except for one PCG (ND6) and eight tRNAs (tRNA-Ala, -Asn, -Cys, -Gln, -Glu, -Pro, -Ser, and -Tyr). Most protein-coding genes start with the ATG codon, with the exception of ND3 and ND5, where they start with ATA. The recognizable complete stop codon TAA or AGA formed the stop codon for eight PCGs, with the other five PCGs (ND1-4 and COXIII) exhibiting incomplete stop codons with a single T– and TA- residue, respectively. The 22 tRNA genes ranged in length from 60 to 75 bp. The length of D-loop is 892 bp, which between tRNA-Phe and tRNA-Pro.

Phylogenetic trees were reconstructed using BI and ML analyses, based on the nucleotide dataset (13 PCGs). The best-fit TPM2uf + G model was selected in jModelTest 0.1 (Darriba et al. 2012), and yielded identical phylogenetic trees by high node-supporting values, including that 29 reported Bovinae species (Figure 1). It showed that *B. grunniens* breed Hongyuan and *B. grunniens* breed Qinghai Plateau converged on the same branch and they have a close genetic relationship. In conclusion, our study characterized the complete mitogenome of *B. grunniens* breed Hongyuan, and determined its systematic classification status, which would contribute to the management and molecular breeding of yak breeds, analysis of the molecular evolution and genetic structure of bovines, and protection of genetic resources.

**Figure 1.**
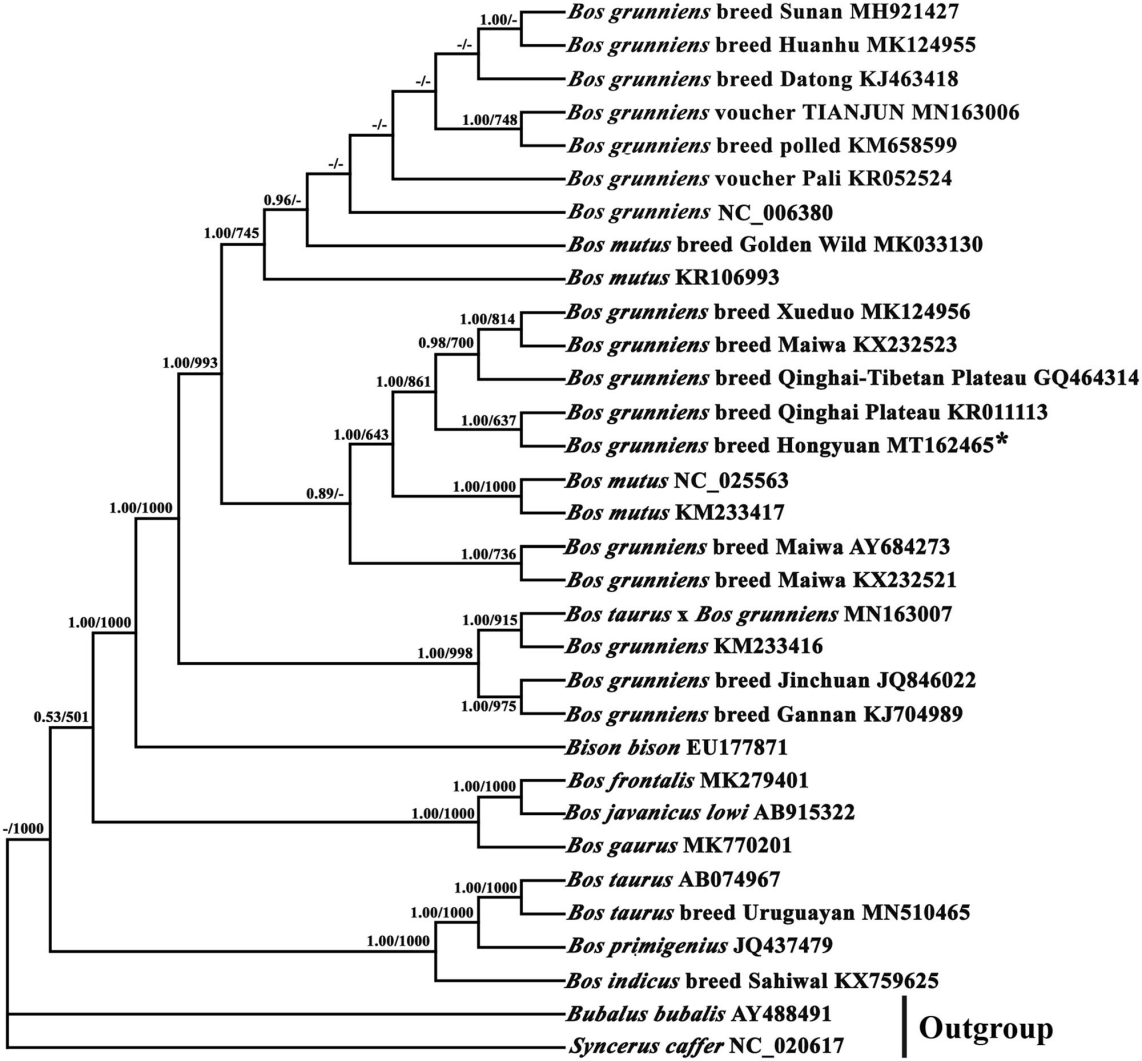
Phylogenetic relationship of mitochondrial genomes of 30 close species and *Bubalus bubalis* and *Syncerus caffer* as outgroups based on the nucleotide dataset of the 13 mitochondrial protein-coding genes. Branch lengths and topology are from the ML analysis. Numbers above branches specify posterior probabilities from Bayesian inference (BI) and bootstrap percentages from maximum likelihood (ML, 1000 replications) analyses. Tree topologies produced by Bayesian inferences (BI) and maximum likelihood (ML) analyses were similar. Bayesian posterior probability and bootstrap support values for ML analyses are shown orderly on the nodes and the symbol "-" indicates that the posterior probability is less than 0.5 or bootstrap support value is less than 500. The asterisks indicate new sequences generated in this study.

## Data Availability

The data that support the findings of this study are openly available in GenBank of NCBI at https://www.ncbi.nlm.nih.gov, accession number MT162465.

## References

[CIT0001] Bao P, Guo X, Pei J, Liang C, Ding X, Min C, Wang H, Wu X, Yan P. 2016. Complete mitogenome sequencing and phylogenetic analysis of PaLi yak (*Bos grunniens*). Mitochondrial DNA A DNA Mapp Seq Anal. 27(6):4590–4591. 27207722 10.3109/19401736.2015.1046128

[CIT0002] Cheng YZ, Xu TJ, Jin XX, Wang RX. 2011. Complete mitochondrial genome of the yellow drum *Nibea albiflora* (Perciformes, Sciaenidae). Mitochondrial DNA. 22(4):80–82. 10.3109/19401736.2011.624602 21985406

[CIT0003] Chu M, Wu X, Liang C, Pei J, Ding X, Guo X, Bao P, Yan P. 2016. The complete sequence of mitochondrial genome of polled yak (*Bos grunniens*). Mitochondrial DNA A DNA Mapp Seq Anal. 27(3):2032–2033. 25347693 10.3109/19401736.2014.974175

[CIT0004] Darriba D, Taboada GL, Doallo R, Posada D. 2012. JModelTest 2: more models, new heuristics and parallel computing. Nat Methods. 9(8):772. 10.1038/nmeth.2109 PMC4594756 22847109

[CIT0005] Douglas KC, Halbert ND, Kolenda C, Childers C, Hunter DL, Derr JN. 2011. Complete mitochondrial DNA sequence analysis of Bison bison and bison-cattle hybrids: function and phylogeny. Mitochondrion. 11(1):166–175. 20870040 10.1016/j.mito.2010.09.005

[CIT0006] Fu D, Ma X, Jia C, Lei Q, Wu X, Chu M, Ding X, Bao P, Pei J, Guo X, et al. 2019. The complete mitochondrial genome sequence and phylogenetic analysis of Maiwa Yak (*Bos Grunniens*). Mitochondrial DNA B. 4(1):1986–1987.

[CIT0007] Gu Z, Zhao X, Li N, Wu C. 2007. Complete sequence of the yak (*Bos grunniens*) mitochondrial genome and its evolutionary relationship with other ruminants. Mol Phylogenet Evol. 42(1):248–255. 16942892 10.1016/j.ympev.2006.06.021

[CIT0008] Guo X, Pei J, Bao P, Chu M, Wu X, Ding X, Yan P. 2016. The complete mitochondrial genome of the Qinghai Plateau yak *Bos grunniens* (Cetartiodactyla: Bovidae). Mitochondrial DNA A DNA Mapp Seq Anal. 27(4):2889–2890. 26478258 10.3109/19401736.2015.1060423

[CIT0009] Hassanin A, Delsuc F, Ropiquet A, Hammer C, Jansen van Vuuren B, Matthee C, Ruiz-Garcia M, Catzeflis F, Areskoug V, Nguyen TT, et al. 2012. Pattern and timing of diversification of Cetartiodactyla (Mammalia, Laurasiatheria), as revealed by a comprehensive analysis of mitochondrial genomes. C R Biol. 335(1):32–50. 22226162 10.1016/j.crvi.2011.11.002

[CIT0010] Hu Q, Ma T, Wang K, Xu T, Liu J, Qiu Q. 2012. The yak genome database: an integrative database for studying yak biology and high-altitude adaption. BMC Genomics. 13(1):600. 23134687 10.1186/1471-2164-13-600 PMC3507758

[CIT0011] Ma Z, Song H, Zhong J, Stanton D, Wei Y, Sun Y. 2012. Short communication: molecular characterisation of the wild yak (*Bos grunniens* mutus) melanocortin receptor-4 (MC4Y) gene and a comparative analysis with that of other bovinae species. J Appl Anim Res. 40(2):81–85.

[CIT0012] Sambrook J, David WR. 2001. Molecular cloning: a laboratory manual. 3rd ed. New York: Cold Spring Harbor Laboratory Press.

[CIT0013] Tu J, Si F, Wu Q, Cong B, Xing X, Yang FH. 2014. The complete mitochondrial genome of the Muscovy duck, *Cairina moschata* (Anseriformes, Anatidae, Cairina). Mitochondrial DNA. 25(2):102–103. 23992245 10.3109/19401736.2013.784756

[CIT0014] Wang Z, Shen X, Liu B, Su J, Yonezawa T, Yu Y, Guo S, Ho SYW, Vilà C, Hasegawa M, et al. 2010. Phylogeographical analyses of domestic and wild yaks based on mitochondrial DNA: new data and reappraisal. J Biogeogr. 37(12):2332–2344.

